# HIV incidence among women engaging in sex work in sub-Saharan Africa: a systematic review and meta-analysis

**DOI:** 10.1101/2023.10.17.23297108

**Published:** 2023-10-18

**Authors:** Harriet S Jones, Rebecca L Anderson, Henry Cust, R. Scott McClelland, Barbra A. Richardson, Harsha Thirumurthy, Kalonde Malama, Bernadette Hensen, Lucy Platt, Brian Rice, Frances M Cowan, Jeffrey W. Imai-Eaton, James R Hargreaves, Oliver Stevens

**Affiliations:** 1 Faculty of Public Health and Policy, London School of Hygiene and Tropical Medicine, London, UK; 2 MRC Centre for Global Infectious Disease Analysis, School of Public Health, Imperial College London, London, United Kingdom; 3 Department of Global Health and Development, London School of Hygiene and Tropical Medicine, London, UK; 4 Department of Medicine, University of Washington, Seattle, WA, USA; 5 Department of Epidemiology, University of Washington, Seattle, WA, United States of America; 6 Department of Biostatistics, University of Washington, Seattle, WA, USA; 7 Department of Global Health, University of Washington, Seattle, WA, United States of America; 8 Department of Medical Ethics and Health Policy, University of Pennsylvania, Philadelphia, Pennsylvania, USA; 9 University of Toronto Factor-Inwentash Faculty of Social Work, Toronto Ontario, Canada; 10 Sexual and Reproductive Health Group, Department of Public Health, Institute of Tropical Medicine, Antwerp, Belgium; 11 Sheffield Centre for Health and Related Research (SCHARR); School of Medicine and Population Health, University of Sheffield, UK; 12 Department of International Public Health, Liverpool School of Tropical Medicine, Liverpool, UK; 13 Centre for Sexual Health and HIV/AIDS Research Zimbabwe, Harare, Zimbabwe; 14 Center for Communicable Disease Dynamics, Department of Epidemiology, Harvard T. H. Chan School of Public Health, Boston, MA, USA

## Abstract

**Introduction:**

HIV incidence among women in sub-Saharan Africa (SSA) has declined steadily, but it is unknown whether new infections among women who engage in sex work (WESW) have declined at a similar rate. We synthesised estimates of HIV incidence among WESW in SSA and compared these to the wider female population to understand levels and trends in incidence over time.

**Methods:**

We searched Medline, Embase, Global Health, Popline, Web of Science, and Google Scholar from January 1990 to October 2022, and grey literature for estimates of HIV incidence among WESW in SSA. We included studies reporting empirical estimates in any SSA country. We calculated incidence rate ratios (IRR) compared to age-district-year matched total female population incidence estimates. We conducted a meta-analysis of IRRs and used a continuous mixed-effects model to estimate changes in IRR over time.

**Results:**

From 32 studies between 1985 and 2020, 2,194 new HIV infections were observed in WESW over 51,000 person-years (py). Median HIV incidence was 4.3/100py (IQR 2.8–7.0/100py), declining from a median of 5.96/100py between 1985 and 1995 to a median of 3.2/100py between 2010 and 2020. Incidence among WESW was nine times higher than in matched total population women (RR 8.6, 95%CI: 5.7–12.9), and greater in Western and Central Africa (RR 22.4, 95%CI: 11.3–44.3) than in Eastern and Southern Africa (RR 5.3, 95%CI: 3.7–7.6). Annual changes in log IRRs were minimal (−0.1% 95%CI: −6.9 to +6.8%).

**Conclusions:**

Across SSA, HIV incidence among WESW remains disproportionately high compared to the total female population but showed similar rates of decline between 1990 and 2020. Improved surveillance and standardisation of approaches to obtain empirical estimates of sex worker incidence would enable a clearer understanding of whether we are on track to meet global targets for this population and better support data-driven HIV prevention programming.

## Introduction

New HIV infections have steadily declined among women in sub-Saharan Africa (SSA) since 1994, including by 65% between 2010–2022 in Eastern and Southern Africa (ESA).^1^ However, among women across SSA, those who engage in sex work bear a disproportionate burden of HIV.^1,2^ Women who engage in sex work (WESW) comprise an estimated 1.2% of woman aged 15–49 in SSA, but an estimated 3.5% of women living with HIV in the region.^2^ HIV prevention programmes for WESW were a core component of the early HIV response in SSA, however reduced funding and a shift to general population programming meant a decline in programmes focused on WESW from the early 2000s.^3^ More recently, renewed energy in targeted approaches for key populations has resulted in the re-expansion of programming for WESW.^4–6^ Assessing the success of these efforts on preventing new HIV infections is challenging, and it is unknown whether HIV incidence among WESW has declined in line with women in the wider population.

Despite increasing surveillance and programmatic support for WESW, studies of HIV incidence in SSA remain infrequent and challenging. Identifying and following-up WESW is often difficult due to the heterogeneous, informal, and hidden nature of sex work, commonly driven by stigma and criminalisation.^7^ For these reasons WESW are largely unidentified in population-based surveys and constructing a national sampling frame usually impractical. Sex work encompasses a broad spectrum of sexual transactions occurring in a range of settings, from on the streets, to in homes, brothels, or hotels.^8^ Women who engage in transactional sex may not self-identify as sex workers. For surveillance, this presents challenges, as those not identifying as sex workers are unlikely to present at WESW-dedicated programmes. Additionally, mobility among WESW is high,^9^ and repeated initiation and cessation of sex work common, thus programmes and cohort studies encounter challenges with loss to follow-up.^10,11^ To mitigate these challenges, there is growing focus on techniques leveraging cross-sectional data to estimate incidence,^12,13^ whilst recruitment approaches such as respondent-driven sampling (RDS) and time-location sampling (TLS) are increasingly used to capture more representative samples of WESW.

As countries seek to reach the global goal of ending AIDS as a public health threat by 2030 through ending inequalities, quantifying HIV incidence trends in WESW is required to guide national HIV programme planning and delivery. In this study we aimed to synthesise and appraise empirical estimates of HIV incidence in WESW in SSA, estimate relative HIV incidence between WESW and the total female population for WCA and ECA, and estimate the change in relative HIV incidence over time.

## Methods

We searched published and grey literature to identify empirical estimates of HIV incidence among WESW in SSA. We conducted two searches of peer-reviewed literature available in English and published between January 1990 and December 2022. Medline, EMBASE, Popline, Web of Science, and Global Health were searched on 19^th^ June 2019 using medical subject headings (MeSH) and text words adapted for each database covering three domains: ‘Female sex workers’, ‘HIV’, and ‘sub-Saharan Africa’. Searches were updated in January 2023 in Medline, EMBASE, Global Health, and Google Scholar using text words addressing four domains: ‘sex workers’, ‘sub-Saharan Africa’, ‘HIV’ and ‘incidence’ (Supplementary Text S1). The initial title, abstract, and full text screening was conducted by HSJ and HC in 2019 for an earlier review of HIV testing among WESW^14^ and full texts subsequently searched for those reporting HIV incidence. A second search was conducted by RLA in January 2023, with all papers selected for final inclusion based on consensus between HSJ, RLA, and OS. RLA additionally searched key population biobehavioural surveillance reports, collated during an earlier key population data collation exercise.^2^

### Study inclusion

**Figure 1:**
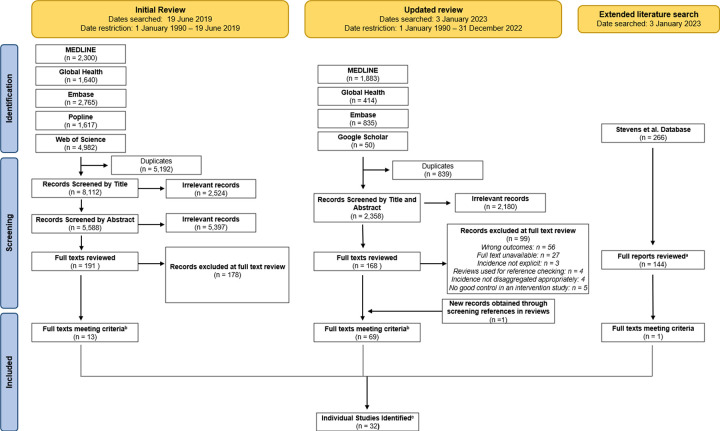
Flow diagram summarising identification, screening, and inclusion of studies. a: Reports retrieved through key term search for the word “incidence” in File Explorer. b: **Full texts meeting criteria** include multiple papers reporting on the same study. c. **Individual Studies Identified** are the primary texts which data were extracted from.

For inclusion, studies needed to report an empirically measured estimate of HIV-test confirmed HIV incidence in WESW in any SSA country or report the number of HIV events and total person-years (py) at-risk to manually calculate incidence. WESW could be women either self-reporting being a sex worker, engaged in a sex worker programme, or reporting selling or exchanging sex for money or goods. For multi-country studies, nationally disaggregated data were required. For closed cohort studies reporting annual estimates, only the overall estimate of incidence from the study was extracted to mitigate against artificial declines in incidence observed with follow-up of the same individuals. Studies were excluded if the study cohort was exposed to an intervention specifically thought to have impacted HIV incidence, i.e. HIV pre-exposure prophylaxis (PrEP). In randomised control trials (RCT), data from the study control arm were included and, in interventional cohort studies, data from early in the intervention or pre-intervention period included. Conference abstracts or published short communications were excluded. Where multiple papers reported incidence estimates for the same study population, the paper reporting the greatest number of person-years of follow-up was selected as the primary study for data extraction (Figure 1).

### Data extraction

From each study, two authors extracted the study location (country, subnational area), time period, study population definition, recruitment strategy, mean/median age and recruitment range, incidence measurement method (e.g. recency testing, repeat HIV tests), study type (e.g. cohort, RCT), sample size, person-years of follow-up, number of seroconversions, incidence per 100py, and confidence intervals, disaggregated by age-group where available. We contacted study authors where estimates were not clearly available in existing publications (n=1),^15^ not disaggregated to WESW (n=1),^16^ or from studies reporting data for extended time periods which could provide annual estimates to assess temporal trends (n=2).^17,18^

### Quality assessment

We appraised studies using the Global HIV Quality Assessment Tool for Data Generated through Non-Probability Sampling (GHQAT).^19^ The tool comprises three domains: study design, study implementation and a measurement specific domain for HIV incidence. Domain specific assessment criteria are in the tool and supplementary material (Supplementary Table 2). We defined scoring requirements for subjective assessment criteria, stipulating reporting requirements needed to score positively. Measurement quality scored positively if studies stated their approach to seroconversion date measurement. Sample recruitment scored positively if studies used a network sampling approach (e.g. RDS^20^), TLS, or clinic sampling with peer recruitment. Where physical locations were used for recruitment, location mapping needed to be reported. Studies were also scored positively if study length was at minimum 12 months, retention >70%, tracing was conducted for participants lost-to-follow-up or statistical adjustments made to account for loss-to-follow-up. We chose not to score studies on overall reporting quality. Based on a score of 1 if studies met the assessment criteria and 0 if they did not, each was classified as good, fair, or poor for each domain, and then overall. Studies scoring ≥ 70% were classified as good, between 30% and 70% fair and <30% poor. We adapted scoring to account for exclusion of reporting criteria and for studies calculating incidence from cross-sectional recent infection testing or serial HIV prevalence measures.^12,13^

### Data synthesis and meta-analysis

HIV incidence observations among WESW were matched to HIV incidence in the total female population by district (subnational location where each study was conducted), age, and year. Estimates missing age information were matched to incidence in total population women aged 15–39, reflecting the age distribution observed in sex workers in studies reporting age data. District-level estimates in 2022 for the total female population were extracted from UNAIDS subnational HIV estimates created using the Naomi model.^21^ Naomi is a district-level small-area estimation model that calibrates to nationally representative household survey and routine ART and antenatal health system data. District-level incidence estimates for 1985–2021 were created by extrapolating 2022 estimates backwards in time, parallel to UNAIDS national-level female incidence trajectories, assuming the proportional change in incidence at district-level mirrored that at national-level.^22,23^ For studies where annual incidence estimates were unavailable, the midpoint study year was used for matching. When a subnational location was not specified in the text (n=3), national total population incidence was used as the comparator. One study presented incidence for a control group of women not engaged in sex work,^24^ which was used as the total population comparator instead of matching to UNAIDS’ district estimate.

We assessed correlation between WESW and matched total population female incidence and calculated incidence rate ratios (IRRs). We used a meta-analysis of IRRs, with study-country-district nested random effects to account for variation in study type, and regional fixed effects to stratify our pooled IRRs by region (ESA and Western and Central Africa (WCA)). We examined trends in IRRs over time using a Bayesian mixed-effects log-linear model, predicting log-IRRs using calendar year fixed effects, study-level random effects, and total female population incidence and person-years of follow-up as offsets (Supplementary Text S2). Lastly, we conducted case studies for the two countries with the most available data. We used data from an open cohort in Mombasa, Kenya,^18^ and from a national key populations programme in Zimbabwe run by the Centre for Sexual Health and HIV/AIDS Research Zimbabwe (CeSHHAR),^17^ to descriptively assess temporal trends between WESW and total female population incidence.

We conducted several sensitivity analyses. Firstly, to address uncertainty surrounding the district-level total population incidence estimates, we repeated the meta-analysis using national age-sex matched population incidence as the denominator for IRRs. To assess the impact of study quality, we repeated the meta-analysis filtered to only higher quality studies (those scoring 60% or above on the GHQAT). Finally, as the majority of empirical incidence estimates for WESW were from populations in Kenya and Zimbabwe, we refit the mixed-effects model to data from both countries. Analyses were implemented in R v4.2.1^25^ using the *metafor* v3.8.1^26^ and *R-INLA* v22.5.7^27^ packages.

### Ethical approval

This study received ethical approval from the Imperial College Research and Ethics Committee (#6412027). For use of CeSHHAR’s Key Populations programme data, ethical approval was obtained from the London School of Hygiene and Tropical Medicine (16543) and the Medical Research Council of Zimbabwe (MRCZ/A/2624).

## Results

We extracted 83 estimates of HIV incidence among WESW in sub-Saharan Africa from 32 studies reported in 69 peer-reviewed papers and one surveillance report (Tables 1 and 2). The majority of estimates were from ESA (78%; 65/83), predominantly from Kenya and Zimbabwe (59%, 49/83). In WCA, 18 incidence estimates were reported from eight countries. Median study year was 2008 (IQR: 2000–2015). Between 1985 and 2020, 2,194 new HIV infections were observed from 51,000py with median HIV incidence of 4.3/100py (interquartile range [IQR] 2.8–7.0/100py).

Over half the studies were cohort studies (59%; 19/32), one fifth were randomised control trials or intervention studies (22%; 7/32), five were cross-sectional studies (16%, 5/32), and one study used routine clinic data (1/32; Table 2). Study populations included women who self-identified as sex workers (7/32), exchanged sex for money or goods (13/32), either over a defined period or as a primary or secondary source of income, or worked in a known sex work location (2/32). Six study populations were women linked to clinics or sex worker programmes. Study reach varied widely, from studies in single clinic populations (6/32) to single towns, cities, or regions (19/32), or multiple locations nationally in South Africa and Zimbabwe (8/32). Recruitment methods included network sampling approaches (7/32), time location sampling (4/32), clinic recruitment (7/32), or convenience samples from peer outreach activities or community meetings (9/32). Study participant ages ranged from a median of 15 (IQR 14–17)^28^ to a mean of 38^29^ with 11/32 studies reporting a mean or median age between 25 and 30. Incidence estimates were predominantly derived from inference of a seroconversion date between HIV tests (26/32). Most of these studies used the midpoint between first positive and last negative test result (13/25), two used the HIV-positive test date, and seven did not report a clear seroconversion date estimation approach. Four studies estimated incidence from recency assays, and two used back calculation from age-specific HIV prevalence.

### Quality assessment

Assessment with the GHQAT classified 8/32 (25%) studies as ‘good’ quality, and the remaining 24/32 (77%) as ‘fair’ (Supplementary Table S2). Studies all scored well in terms of study design, with study objectives and study populations clearly defined. Most studies either had robust approaches to sampling or did not seek to generalise their findings beyond their study population so were considered adequately representative (26/32). Power calculations were presented in one-third of studies (9/32). Scores under study implementation varied, with only 10/31 studies reporting participation rates, of which six reported over 85% participation. For 19/32 studies, it was likely that participants enrolled were representative of the source population. Scoring for measurement of HIV incidence was variable. Among longitudinal studies, 12 report >70% participant retention at 12 months or study endpoint. Only five described methods to address loss to follow up.

### Data synthesis & meta-analysis

**Figure 2:**
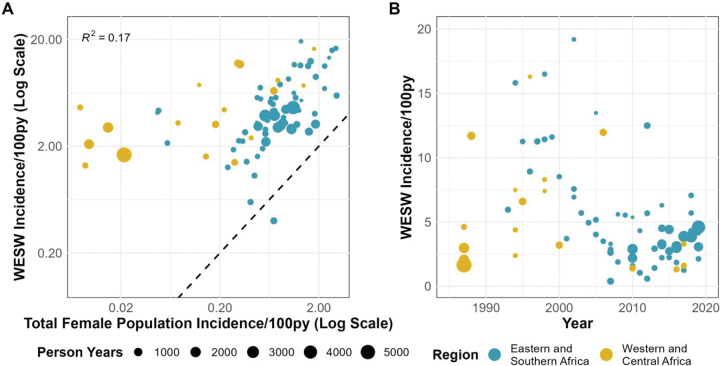
Empirical estimates of HIV Incidence among women engaging in sex work. A) Association between HIV incidence in sex workers and in the district-year-sex matched total population. B) Empirical estimates of HIV incidence in sex workers over time. Black dashed line in A represents the line of equality; WESW = women who engage in sex work; py = person-years

Incidence among WESW was correlated with matched female incidence (R = 0.41; Figure 2A). In meta-analysis, the HIV incidence rate in WESW was nine times higher than in matched total population women (RR 8.6, 95%CI 5.7–12.9; Figure 1). Rate ratios were greater in WCA (RR 22.4, 95%CI 11.3–44.3) than in ESA (RR 5.3, 95%CI 3.7–7.6). The sensitivity analysis using the 21 studies scoring above 60% through quality assessment yielded a pooled IRR of 6.9 (95% CI 4.4–10.8, Supplementary Figure 1). Sensitivity analysis using nationally-matched incidence increased relative risk (RR: 10.6, 95% CI 7.4–15.2, Supplementary Figure 2).

**Figure 3:**
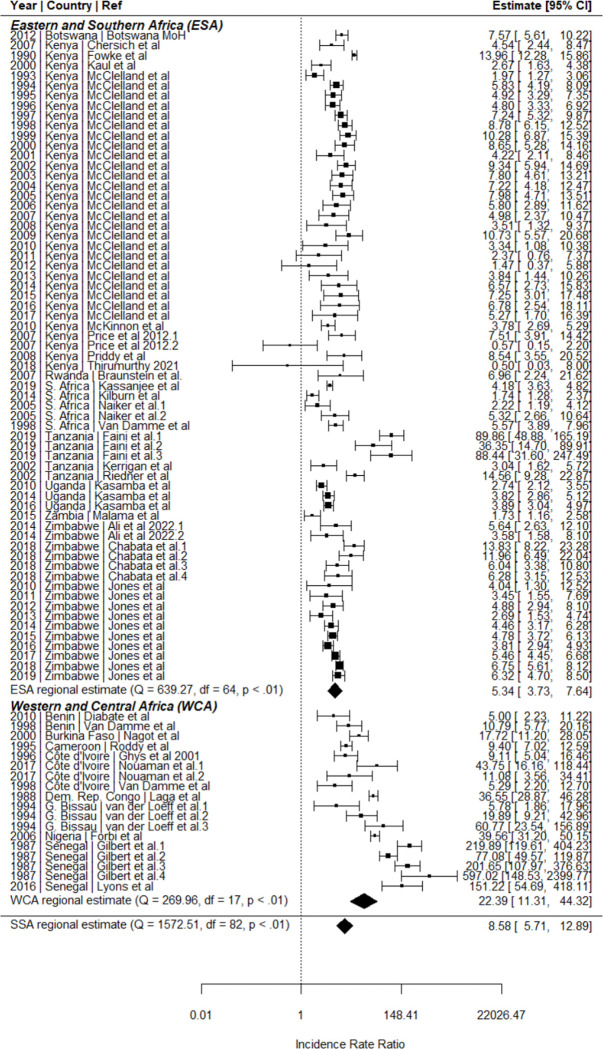
Meta-analysis of HIV incidence in women engaging in sex work relative to the total female population in Sub-Saharan Africa. IRRs calculated by dividing empirical estimates of sex worker HIV incidence by HIV incidence among age-district-year matched total population women derived from the district-level estimation model Naomi^21^. ESA: Eastern and Southern Africa; WCA: Western and Central Africa; SSA: Sub-Saharan Africa.

HIV incidence in WESW halved between 1985–1995 and 2010–2020, from a median of 5.96/100py (IQR 3.0–11.3/100py) to median 3.2/100py (IQR 2.2–4.3/100py; Figure 2B). Compared to matched female population incidence, there was no evidence for a change in IRR over time (Odds ratio [OR] 1.00, 95%CI 0.93–1.07; Figure 3). Incidence in WESW was nearly 6 times higher than in the total female population in 2003 according to the mixed-effects model (IRR 5.5, 95% CI 3.8–8.7; Figure 3, Supplementary Table S2).

**Figure 4:**
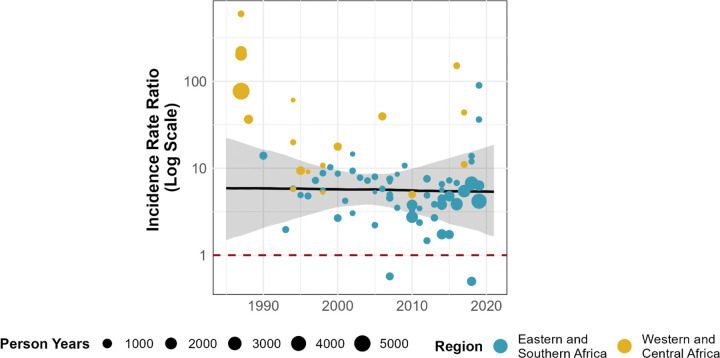
HIV incidence rate ratios modelled over time, presented on the logarithmic scale. Points represent incidence rate ratios calculated by dividing study-reported HIV incidence in women engaging in sex work by age-district-year matched total population HIV incidence derived from the Naomi model^21^. Black line represents the estimated incidence rate ratio (IRR) for Sub-Saharan Africa, with the grey shading capturing the 95% uncertainty range. The red dashed line represents an IRR of 1 (HIV incidence in sex workers = total female population HIV incidence).

In separate model fits to data from Kenya and Zimbabwe, log IRR did not change over time in Kenya (OR 1.01, 95% CI: 1.00–1.03), but increased in Zimbabwe between 2009 and 2019 (OR 1.11, 95% CI 1.05–1.16; Supplementary Table S1). Two studies from these countries provided annual incidence estimates over their follow up-period (Figure 4). In the Kenyan cohort, incidence in WESW peaked at 16/100py in 1994 and declined to 1/100py in 2017. However, the declines in incidence in WESW were matched by reductions in total female population incidence, resulting in a stable IRR over the cohort period. In the Zimbabwean cohort, the HIV incidence rate in WESW also declined from 5/100py to 3/100py from 2010–2019, but the IRR increased due to faster relative declines in total population female incidence.

## Discussion

In sub-Saharan Africa, HIV incidence among women who engage in sex work was nearly nine times greater than in the wider female population. This disparity was larger in WCA, where incidence was almost 23 times higher than in all women, compared to six times higher in ESA. Between 1985 and 2020, incidence in WESW declined at a similar rate to incidence in age-location-year matched total population women, however case studies of Kenya and Zimbabwe illustrated that these trends may vary between countries. Sensitivity analyses using national incidence estimates and restricted to high-quality studies did not alter the interpretation of our findings.

Our analysis builds on existing evidence that WESW are disproportionately impacted by HIV. Our large IRR echoes the five-fold discrepancy reported in HIV prevalence between WESW and the wider population in sub-Saharan Africa.^2^ We additionally estimated a greater relative risk in lower HIV prevalence epidemics in WCA compared to ESA. The declining HIV incidence among WESW found in this analysis was aligned with modelling studies from WCA that estimate the proportion of total new HIV infections attributable to commercial sex has fallen over time.^30,31^ These parallel incidence declines are in contrast with a systematic review of incidence among men who have sex with men in sub-Saharan Africa, which found no such evidence.^32^

Studies varied in their geographic reach, and their definition and recruitment of WESW. Inclusion criteria often defined the age range and duration or frequency of selling sex, or sought women self-identifying as WESW, leading to a range of study populations with likely varying degrees of HIV acquisition risk. Studies among women linked to targeted programmes or clinics may have recruited women at lower risk of HIV acquisition, who may be older or had sold sex for longer durations, potentially missing younger women, those yet to identify as selling sex and in the highest risk period immediately following sex work initiation.^33^ Preliminary findings from South Africa suggest that sex work dynamics are changing with the mean age of sex workers increasing and longer durations at-risk,^34^ whilst young WESW remain at highest incidence risk.^35^ Different sampling and recruitment approaches were also likely to identify different women for study inclusion. Studies have shown that participants recruited through RDS are younger than those recruited through venue-based snowball sampling.^36^ Using district-age-matched IRRs in our analysis likely mitigated some of the bias that would have come from geographical and age differences if raw estimates of incidence were analysed independently.

Whilst declining incidence in WESW may in part be a product of incidence trends and increased programme coverage across all women in SSA, it also reflects substantial efforts in roll-out of WESW-dedicated programmes, treatment, and health services in SSA, and rising treatment coverage among men.^1^ Whilst this finding is positive, our study also highlights the need to sustain and continue to expand effective HIV prevention options and treatment services for WESW, given the persistently large disparity in HIV incidence between WESW and the wider female population, particularly in WCA. Counterfactual-based modelling shows that fully meeting HIV prevention and treatment needs of WESW would substantially impact the HIV epidemic in SSA, and that averting HIV transmission associated with sex work should remain a programmatic priority.^6,31^

Our study was subject to limitations. Data were available from fewer than half of SSA countries, with WCA particularly under-represented, and estimates for ESA largely from Kenya and Zimbabwe. This prevented estimation of country-specific IRRs and non-linear time trends and limited the generalisability of our findings. Data were insufficient to estimate pooled age-disaggregated IRRs which may have provided further insights, given evidence of higher incidence among younger WESW.^35^ For all but one study which provided a comparator non-WESW group,^24^ our IRR denominators were based on extrapolations of subnational estimates backwards in time parallel to national female incidence trajectories. Whilst this approach aimed to capture spatial variation over time, there is substantial uncertainty in these subnational incidence denominators, particularly for older studies, which is not reflected in the calculated IRRs. Additionally, given high mobility among WESW,^9^ matching on district boundaries may not yield the most comparable total female population denominator. However, sensitivity analysis using nationally-matched IRRs gave similar results to the district-matched analysis, alleviating concerns around subnational uncertainties.

We appraised studies using the GHQAT. Although we did not quantify changes in study quality over time, it is likely that study quality improved with improvements in HIV testing and sampling methods. Most studies did not make inferences beyond the study population they recruited and scored positively on adequately representing the wider WESW population. However, it is unlikely that many would have been representative had they made these inferences. Ascertaining the degree of bias associated with sample recruitment posed challenges. There is no standardised approach to sampling individuals from key populations, and although respondent driven sampling is widely accepted as a gold standard to achieving a representative sample,^38^ there was variation in implementation and reporting across studies. We excluded studies providing interventions beyond standard of care but were unable to account for wide variation in programme interventions and intensity, or the influence in historical or geographically proximal HIV responses.

Our review highlights that while HIV incidence data are available for WESW populations, geographical gaps remain, and temporal trends difficult to ascertain from WESW estimates alone, particularly at country level. Individual studies are challenging to compare due to substantial heterogeneity in study design and the limited generalisability of findings beyond individual study populations. Standardisation in definitions for WESW and reporting of age ranges may improve comparability of estimates. The application of methodological standards for directly measuring incidence in key populations would also be beneficial. Data gaps could be addressed by incorporating incidence measurements into survey and routine programmatic data analysis via serial cross-sectional prevalence data from biobehavioural surveys and recency testing,^12,13,17,39^ instead of resource-intensive cohort studies. This would increase the number of available estimates to enable country-specific estimation and support real-time data-driven programming.

## Conclusion

Across SSA, HIV incidence in WESW has declined over the past 20 years but substantial inequalities remain, with incidence nine times greater among WESW compared to the wider female population. While declines are currently hard to ascertain from empirical estimates alone, these can be inferred from the limited change in WESW incidence relative to incidence among women in the wider population. Future surveillance activities should fill these gaps, ensuring a more standardised approach to obtaining empirical estimates of WESW incidence with repeat measures in the same populations to guide data-driven HIV prevention and treatment programme planning and ensure incidence continues to decline, and inequalities are addressed.

## Supplementary Material

Supplement 1

## Figures and Tables

**Figure 5:**
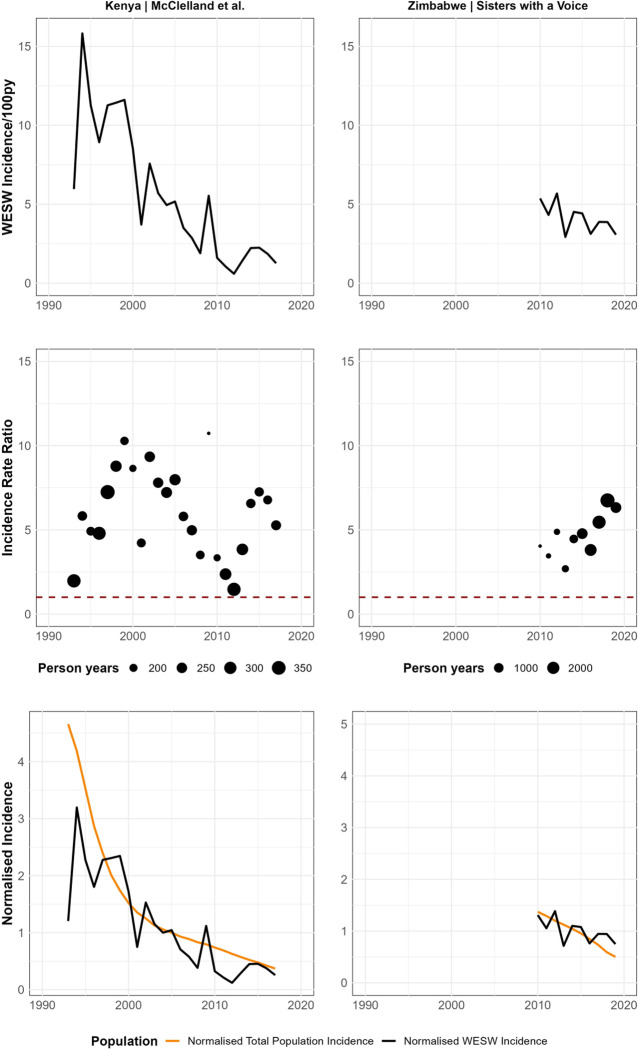
Case Studies - Incidence and Incidence Rate Ratio (IRR) trends over time in data from Mombasa, Kenya (McClelland et al) and Zimbabwe (Jones et al). Upper row: Empirical incidence in women engaging in sex work over time. Middle row: Incidence rate ratio over time. The red dashed line represents an IRR of 1 (HIV incidence in sex workers = total female population HIV incidence). Lower row: Normalised incidence over time. Calculated as a ratio of annual HIV incidence divided by incidence in the median year (1993–2018 for McClelland et al: median year = 2005; 2010–2019 for Jones et al: median year = 2015). WESW: Women who engage in sex work; IRR: Incidence Rate Ratio.

**Table 1:** Annual incidence estimates for women who engage in sex work (WESW) and total population women aged 15–39

Study^a^	Country	Study Period	Area^b^	Mid-point Year	Age Group^c^	WESW New HIV Infections	Person-years (py) of follow-up	WESW Incidence/100py	Total female population incidence/100/py	IRR^d^

Ali (2020)^e^	Zimbabwe	2011–2017	Zimbabwe	2014	15–24	7	105	6.3	1.12	5.64

					25–39	6	175	3.3	0.92	3.58

Botswana MoH (2013)^f^	Botswana	2012	Gaborone, Francistown, Kasane	2012	15–39	46	450	12.5 (7.3, 17.1)	1.65	7.57

*Project Ubuzima* Braunstein (2011)	Rwanda	2006–2008	Kigali	2007	15–39	3	91	3.3 (0.0, 7.0)	0.47	6.96

Chabata (2021)	Zimbabwe	2017–2019	Chinhoyi	2018	15–24	16	226	7.1 (4.3, 11.5)	0.51	13.83
			Karoi			11	193	5.7 (3.2, 10.3)	0.48	11.96
			Kwekwe			12	278	4.3 (2.5, 7.6)	0.71	6.04
			Zvishavane			9	210	4.3 (2.2, 8.2)	0.68	6.28

Chersich (2014)	Kenya	2006–2007	Mombasa (Kisauni and Chaani)	2007	15–39	10	381	2.6	0.58	4.54

Diabate (2018)	Benin	2008–2012	Cotonou	2010	15–39	6	425	1.4 (0.3, 2.5)	0.28	5

Faini (2022)	Tanzania	2018–2019	Dar es Salaam	2019	15–24	12	278	4.3	0.05	89.86
					25–34	5	234	2.1	0.06	36.35
					35–44	4	97	4.1	0.05	88.44

Forbi (2011)	Nigeria	2006	Nasarawa State	2006	15–34	71	590	11.97 (8.5, 15.4)	0.3	39.56

Fowke (1996)	Kenya	1985–1994	Nairobi	1990	15–39	239	569	42.0	3.01	13.96

Ghys (2001)	Cote d’Ivoire	1994–1998	Abidjan	1996	15–39	11	68	16.3	1.79	9.11

Gilbert (2003)	Senegal	1985–1999	Dakar	1987	15–24	34	1628	2.1	0.01	219.9
			Dakar		25–34	91	5474	1.7	0.02	77.08
			Dakar		35–44	58	1939	3.0	0.01	201.7
			Dakar		45–49	12	260	4.6	0.01	597

Jones (2023)^g^	Zimbabwe	2010–2019	Zimbabwe	2010	15–39	3	56	5.4 (3.2, 10.9)	1.33	4.04
				2011		6	139	4.3 (2.2, 14.6)	1.25	3.45
				2012		15	264	5.7 (1.9, 22.8)	1.16	4.88
				2013		12	410	2.9 (1.5, 8.1)	1.09	2.69
				2014		33	731	4.5 (2.5, 8.2)	1.01	4.46
				2015		62	1402	4.4 (3.6, 5.4)	0.93	4.78
				2016		58	1857	3.1 (2.5, 4.1)	0.82	3.81
				2017		94	2422	3.9 (3.3, 4.65)	0.71	5.46
				2018		114	2946	3.9 (3.3, 4.7)	0.57	6.75
				2019		44	1432	3.1 (2.1, 5.0)	0.49	6.32

Kasamba (2019)	Uganda	Cohort 1: 2008–2017 Cohort 2: 2013–2017	Kampala	2010	15–39	59	2007	2.9	1.06	2.74
				2014		46	1394	3.3	0.86	3.82
				2016		65	2138	3.0	0.77	3.89

Kassanjee (2022)^f^	South Africa	2019	South Africa	2019	15–49	190	4130	4.6 (1.5, 8.5)	1.1	4.18

Kaul (2004)	Kenya	1998–2002	Nairobi	2000	15–39	16	495	3.2	1.2	2.67

Kerrigan (2017)	Tanzania	2015–2017	Mafinga	2002	15–39	10	144	6.9	2.28	3.04

Kilburn (2018)	South Africa	2012–2015	Mpumalanga	2014	15–19	41	1273	3.2	1.85	1.74

Laga (1994)	DRC	1988–1991	Kinshasa	1988	15–39	70	880	11.7	0.32	36.55

Lyons (2020)	Senegal	2015–2017	Dakar, Mbour, Theis	2016	15–39	4	303	1.3 (0.5, 3.5)	0.01	151.2

Malama (2022)	Zambia	2012–2017	Ndola, Lusaka	2015	15–44	24	884	2.7	1.59	1.73

McClelland (2015)^g^	Kenya	1993–2017	Mombasa	1993	15–39	20	335	6.0	3.02	1.97
				1994		36	228	15.8	2.72	5.83
				1995		24	213	11.3	2.29	4.92
				1996		29	325	8.9	1.86	4.8
				1997		41	364	11.3	1.56	7.24
				1998		31	271	11.4	1.3	8.78
				1999		24	207	11.6	1.13	10.28
				2000		16	188	8.5	0.99	8.65
				2001		8	216	3.7	0.88	4.22
				2002		19	251	7.6	0.81	9.34
				2003		14	245	5.7	0.73	7.8
				2004		13	263	5.0	0.69	7.22
				2005		14	271	5.2	0.65	7.98
				2006		8	228	3.5	0.6	5.8
				2007		7	244	2.9	0.58	4.98
				2008		4	211	1.9	0.54	3.51
				2009		9	163	5.5	0.52	10.73
				2010		3	187	1.6	0.48	3.34
				2011		3	283	1.1	0.45	2.37
				2012		2	334	0.6	0.41	1.47
				2013		4	280	1.4	0.37	3.84
				2014		5	225	2.2	0.34	6.57
				2015		5	222	2.3	0.31	7.25
				2016		4	216	1.9	0.27	6.78
				2017		3	237	1.3	0.24	5.27

McKinnon (2015)	Kenya	2008–2011	Nairobi	2010	15–39	34	1514	2.2 (1.6, 3.1)	0.58	3.78

Nagot (2005)	Burkina Faso	1998–2002	Bobo-Dioulasso	2000	15–39	19	594	3.2 (1.9, 4.9)	0.18	17.72

Naiker (2015)	South Africa	2004–2006	Durban	2005	25–49	10	248	4.0	1.82	2.22
					15–24	8	59	13.5	2.53	5.32

Nouaman (2022)	Cote d’Ivoire	2016–2017	San Pedro	2017	15–39	4	188	3.3	0.08	43.75
			Abidjan			3	293	1.6	0.14	11.08

Price (2012)	Kenya	2008	Kilifi	2007	15–39	9	339	2.7 (1.4, 5.1)	0.36	7.51
			Nairobi			2	527	0.4 (0.1, 1.5)	0.7	0.57

Priddy (2011)	Kenya	2008	Nairobi (Mukuru District)	2008	15–39	5	89	5.6 (1.6, 12.0)	0.66	8.54

Riedner (2006)	Tanzania	2000–2003	Mbeya Region	2002	15–39	19	99	19.2	1.32	14.56

Roddy (1998)	Cameroon	1994–1996	Yaounde, Douala	1995	15–39	46	698	6.6	0.7	9.4

Thirumurthy (2021)^g^	Kenya	2017–2020	Siaya County	2018	15–39	0	939	0.0	0.41	0

Van Damme (2002)	South Africa	1996–2000	KwaZulu-Natal	1998	15–39	30	182	16.5	2.96	5.57
	Benin		Cotonou			10	121	8.3	0.77	10.79
	Cote d’Ivoire		Abidjan			5	67	7.4	1.4	5.29

Van der Loeff (2001)	Guinea-Bissau	1989–1998	Guinea-Bissau	1994	15–39	3	126	2.4	0.41	5.78
					40–49	7	160	4.4	0.22	19.89
					50–59	5	67	7.5	0.12	60.77

a. Referenced study taken as the primary study from which estimates extracted from, or from whom unpublished estimates were received.

b. Area used to matching WESW incidence to total female population incidence estimates to calculate incidence rate ratios.

c. Age group used to match WESW incidence to total female population incidence estimates; when no age information was specified a default of 15–39 years was used.

d. Incidence Rate Ratios (IRR) calculated by dividing study-reported WESW incidence by area-year matched total population female incidence. Total population female incidence in 2022 extracted from Naomi, the UNAIDS-supported district-level estimation model^21^, and extrapolated parallel to national-level female HIV incidence trajectories from UNAIDS Global HIV Estimates 2022, National Spectrum estimates^22,23^.

e. Incidence estimate derived from serial cross-sectional prevalence testing with no estimate of person-years or number of new infections. The number of person-years was imputed using the median number of person-years across all studies and split proportionally according to the denominator in each age group. Imputed person-years values were then multiplied by the study-reported HIV incidence to back-calculated the number of HIV infections.

f. Incidence estimate derived from recent infection testing with a mean duration of recent infection of six months. The number of person-years was derived by multiplying the number of tested individuals by 0.5.

g. Unpublished WESW incidence and person-years of follow-up obtained through personal communications with study authors.

IRR = incidence ratio ratio; WESW = female sex worker; py = person-years; DRC = Democratic Republic of Congo

**Table 2:** Study characteristics

Study	Country	Age	Definition of women who engage in sex work (WESW)	Study design & recruitment	Incidence estimation	Additional papers identified
Ali (2020)^12^	Zimbabwe	18–39	WESW that had exchanged sex for money in the past 30 days and had been living or working in the survey site for at least 6 months	Cross-sectional respondent-driven sampling surveys conducted between 2011–2017 at sex-work hotspots	Prevalence back calculation, pooling data from RDS surveys. Estimation of HIV incidence from analysis of HIV prevalence patterns	
Braunstein (2011)^40^	Rwanda	Median: 24, Range: 18–46	Women who had exchanged sex for money at least once in the last month and/or currently having sex with multiple partners plus having sex at least twice per week (all enrolled women self-reported sex workers)	Cohort recruited through community meetings conducted by community mobilisers	Seroconversion at follow-up 6–12 months after baseline survey. Midpoint estimation between last HIV-negative and first-HIV positive test	^41,42^
Botswana MoH (2013)^39^	Botswana	18+	Women who received either money or a gift or incentive in exchange for sexual favours within the past three months	Time-location sampling at hotspots for recruitment to cross-sectional IBBS survey	Recent infection testing algorithm. BED incidence assay	
Chabata (2021)^43^	Zimbabwe	18–24	Young women who had exchanged sex for money and/or material support in the past month, and explicitly stated that sex acts with men would not have happened in the absence of an exchange, and if they were not planning to move from the site within the next 6 months.	Non-randomised ‘plausibility’ evaluation of DREAMS on HIV incidence. respondent-driven sampling with seeds selected from sex-work hotspots identified through a community mapping	Seroconversion at follow-up 24 months post-recruitment. Midpoint estimation between an HIV-negative and HIV-positive test.	



Chersich (2014)^44^	Kenya	16+ Mean: 25.1, SD: 5.2	Women reporting receipt of money in exchange for sex as part of their livelihood in the last 6 months, sexually active in the past 3 months, and not pregnant at the time of enrolment	Cohort recruited in locations with existing community links through long-standing service provision by implementers and peers through snowball sampling	Seroconversion between quarterly follow-ups. Midpoint estimation between an HIV-negative and HIV-positive test	
Diabete (2018)^45^	Benin	≥ 18	Women attending Dispensaire IST, the main clinic dedicated to WESW in Cotonou	Clinic recruited cohort – all women attending invited to participate	Seroconversion between quarterly follow-ups. Midpoint estimation between an HIV-negative and HIV-positive test.	
Faini (2022)^46^	Tanzania	18–45	Women self-identifying as sex workers who resided within Dar es Salaam, reported to have exchanged sexual intercourse for money within the past month, considered themselves to be at increased risk for HIV infection and willing to undergo pregnancy testing.	Respondent driven sampling recruited cohort	Seroconversion follow-up visits at 3, 6, 9, and 12 months. Midpoint estimation between an HIV-negative and HIV-positive test	
Forbi (2011)^47^	Nigeria	18–35	Active WESW living in brothels within Nasarawa state of North Central Nigeria (results show all reported >1 partner/week)	Cross-sectional cohort recruited from brothels	Recent infection testing algorithm. BED assay. Calculation - Hargrove adjustments and Mcwalter and Welte’s correction.	
Fowke (1996)^48^	Kenya		Prostitutes of lower socioeconomic status (from a slum area in Nairobi) who practice prostitution from their home	Cohort recruited from an existing community-based cohort set up in 1985	Seroconversion between follow-ups every 6 months. Midpoint estimation.	
Ghys (2001)^49^	Cote d’Ivoire	Median: 27, IQR: 22–32	WESW attending the Clinique de Confiance, a HIV/STD clinic only available for those who are WESW or their stable sex partners.	Intervention study - peer educator recruited for a survey before recruitment of HIV-negative WESW for the study	Seroconversion between biannual follow-ups. (from: - HIV −ve to HIV-1 seropositive - HIV-2 seropositive to seroreactive to both HIV-2 and HIV-1 - HIV−ve to HIV-2 seropositive). Midpoint estimation.	
Gilbert (2003)^50^	Senegal	Mean: 30.4, Range: 19–56	Registered sex workers (self-identifying SWs were required by government to register and regularly attend a health clinic)	Clinic recruited cohort	Seroconversion between follow-ups every 6 months. Midpoint estimation.	^51–53^
Jones (2022)^17^	Zimbabwe	Median age at first test 27	Women attending programme clinics - predominantly cis-women who self-identify as selling sex.	Routinely collected clinic data from Sisters with a Voice, a national sex worker programme including community outreach	Seroconversion between two tests after first attending the programme. Midpoint estimation.	^54^
Kasamba (2019)^55^	Uganda	18+ (under 18 included if pregnant, had children or provided for their own livelihood)	Women who reported engaging in commercial sex (self-identified WESW or received money, goods, or other favours in exchange for sex) or employed in an entertainment facility. Our analysis only includes outcomes for those whose source of income at follow-up was sex work alone, or sex work and another job (results were available for those who did not undertake sex work, but these were excluded)	Peer recruited cohort at mapped sex work hotspots	Seroconversion between 3-monthly follow-ups. Random estimation with uniform distribution.	^56–60^
Kassanjee (2022)^13^	South Africa	18+ Median: 32, IQR 27–38	Cisgender women who had sold or transacted in sex in the preceding 6 months and worked in one of the districts that were studied.	Cross-sectional respondent driven sampling survey recruitment at hotspots visited by outreach programmes	Recent infection testing algorithm. Kassanjee method for incidence calculation. MDRI 145, FRR 0·50%	
Kaul (2004)^61^	Kenya	18+ Mean: 29.1, SD: 7.8	Women who reported having received money or gifts in exchange for sex over the past month	RCT recruited through a series of community visits assisted by peer educators.	Seroconversion between follow-ups every 6 months.	^62–67^
Kerrigan (2017)^68^	Tanzania	18+ Mean: 27.8	Women who reported exchanging sex for money in the past month.	RCT recruited through time location sampling at entertainment venues	Seroconversion between baseline and follow-up at 18 months.	
Kilburn (2018)^24^	South Africa	Recruited ages 13–20, enrolled in high school. Median 15, IQR: 14–17	Young women who reported transactional sex (where they felt that they had to have sex with a male partner as he gave them money or gifts) with any partner in the past 12 months	RCT recruited through the Agincourt Health and Social Demographic Surveillance System (HDSS). Participants visited at home to check eligibility for enrolment	All participants were assessed before randomization and then reassessed annually at 12, 24, and 36 months until they graduated from high school or the study ended,	^69^
Laga (1994)^97^	Democratic Republic of Congo		Women who self-identified as sex workers	Cohort study (recruitment method not reported)	6-monthly HIV-1 incidence rates were computed assuming that seroconversion had occurred at mid-point between the first positive HIV-1 serological test and the last negative one.	^98^
Lyons (2020)^70^	Senegal	18+ Mean: 38.5, IQR: 30–45	Women aged assigned the female sex at birth and having been engaged in sex work as a primary source of income during the year prior to enrolment.	Cohort recruited through respondent driven sampling with additional purposive sample recruitment	Time to event survival analysis - seroconversion date was the diagnosis date. 4-month follow-up visits	
Malama (2022)^15^	Zambia	18–45	Women who reported currently exchanging sex for money.	Community recruited cohort through peers and health care workers at bars, lodges, and street	First visit 1 month after enrolment, then 2 months later and then quarterly.	
McClelland (2015)^18^	Kenya	18+ Median: 31, IQR: 26–37	Participants self-reported exchanging sex for cash or in-kind payment. The majority of women reported working in bars, where they met local male clients	1993–1997: Clinic recruited cohort with outreach meetings in bars. 1998–2017: Cohort recruited through peer-led community outreach meetings at bars	Seroconversion estimated between monthly follow-ups. For women who became infected with HIV and had a positive VL first measured at/after seroconversion, the date of infection was estimated at midpoint between the last seronegative and first seropositive visit. For women with a detectable VL prior to seroconversion (i.e., HIV RNA was detected but antibodies were not), the date of infection was estimated to be 17 days prior to the positive VL	^11,71–86^
McKinnon (2015)^87^	Kenya		Anyone enrolled at the SWOP-City clinic – a sex worker outreach program offering integrated HIV prevention, care & treatment services	Community recruited cohort at hotspots through peers and health care workers	Seroconversion between quarterly follow-ups.	
Nagot (2005)^88^	Burkina Faso	15–56	Professional sex workers (“seaters” and “roamers”, averaging 18 to 28 clients per week) and non-professional sex workers (waitresses, fruit/beer sellers, students - who did not identify as a sex worker but reported an average of 2–3 clients per week	Community recruited cohort at workplaces through peers	Seroconversion during follow-up visits which took place every 3 months	
Naicker (2015)^89^	South Africa	18+	Self-identifying sex workers	Purposively recruited cohort through community liaison partners	Seroconversion during monthly follow-ups. Midpoint estimation.	^90^
Nouaman (2022)^91^	Cote d’Ivoire	18+ Median: 25, IQR: 21–29	WESW working at a site of prostitution at the time of the study.	Cross-sectional convenience sample recruitment by CBO staff	Recent infection testing algorithm. MDRI 0.3 years, FRR 0.013	
Price (2012)^92^	Kenya	Median:25, Range: 18–65	Women who had received goods or money for sex	Cohort recruitment through hotspots, VCT centres and peer recruitment	Seroconversion between quarterly follow-ups.	
		Median: 28, Range: 18–59				
Priddy (2011)^93^	Kenya	Mean: 28, Range: 18–55	Women age 18–60, HIV negative, not pregnant, and who reported exchanging sex for money or gifts at least three times in the past month.	Cohort recruited HIV-negative women who attended education sessions for female sex workers in the Mukuru neighbourhood of Nairobi	Seroconversion at follow-up 6 months after baseline.	
Riedner (2006)^94^	Tanzania	16–39	Women working in modern and traditional bars, guesthouses and hotels.	Cohort recruited from project sites (seem to be hotspots)	Seroconversion between 3-monthly follow-ups. Midpoint estimation.	
Roddy (1998)^99^	Cameroon	18–45 Mean: 26	Female sex workers residing in Yaoundé or Douala, Cameroon, who averaged at least four sexual partners per month	RCT recruitment not clear	Seroconversion at yearly follow-up	
Thirumurthy (2021)^16^	Kenya	18+ Median 25, IQR: 22–31	Women who reported sex work as their primary or secondary source of income with ≥2 male sexual partners in the previous 4 weeks, owning or having access to a mobile phone, intending to remain in the study area for 24 months and not being enrolled in another prevention study	Cohort recruited through random sampling in beach and hotspot clusters from a list of all eligible WESW	Seroconversion between 6-monhtly follow-ups.	
Van Damme (2002)^95^	Benin, Cote d’Ivoire, South Africa	18+ (16+ South Africa)	Participants are not users of intravenous drugs or intravaginal spermicides other than the study drug; not pregnant or no wish to become pregnant in the next 6 months	RCT clinic recruitment	Seroconversion between follow-ups occurring every 2 months. Midpoint estimation.	

						^96^
